# Our Book Table

**Published:** 1885-04

**Authors:** 


### OUR BOOK TABLE.

The Principles and Practice of Dentistry, *including Anatomy, Physiology, Pathology, Therapeutics, Dental Surgery and Mechanism.* By Chapin A. Harris, M. D., D. D. S. Eleventh Edition. Revised and edited by Ferdinand J. S. Gorgas, A. M., M. D., D. D. S. Philadelphia: P. Blakiston, Son & Co., 1885.

The publication of the eleventh edition of the oldest of our standard text-books demands something more than a passing notice. It marks an era in our professional literature. We use the term advisedly and after due consideration, for the last decade has witnessed such an advance in the knowledge upon which an intelligent practice is founded, that its first crystallization into our permanent literature may well be considered as a distinctive epoch.

To Chapin A. Harris is universally conceded the position of Father of Modern American Dentistry. He did more to give it its distinctive character, to mark out its future course, to mould and fashion it into what it is to-day, than any other man. That American Dentistry differs from the dentistry of any other country, is mainly due to his writings and teachings. When medical schools were closed against the teaching of dental science, Harris, with his coadjutors, organized an independent college, instituted a new and peculiar degree, and launched dentistry in its own bark. Under the peculiar circumstances of the time there was no other course to take, if we were not to degenerate into mere handicrafture.

The first edition of The Principles and Practice of Dentistry was published by Dr. Harris in 1839, the same year in which he founded *The American Journal of Dental Science*, the first dental journal published, and one year before he established the first dental college in the world. It was a thin, octavo volume, with but a few illustrations, but it very fairly represented the then infant science of dentistry. Although conducting a large private practice, he was constantly engaged in literary work. The publishing of books and journals was not then reduced to a kind of science, as it is to-day, and for many years Dr. Harris was his own publisher. He conducted his private practice, wrote and published books, and edited, and a part of the time published, a monthly journal. How would he have regarded some of the men of to-day, who, with a practice no larger than his own, and nothing else to occupy their time, complain that they have not the necessary leisure for even reading dental journals, to say nothing of a comprehensive study of the literature of their profession.

The second edition of one thousand copies of this work was, by agreement with Dr. Harris, published in Philadelphia, in 1844, by Lindsay & Blakiston, the predecessors of the present publishers. This edition contained many new illustrations, and was considerably increased in size and comprehensiveness. The third edition, of the same number of copies, was published in 1847. The fourth edition, of one thousand copies, was published in 1850. The fifth, in 1852; the sixth, in 1855; the seventh, in 1858; the eighth, in 1863; the ninth in 1866. Each of the last five of these editions consisted of fifteen hundred copies, and each was more or less revised and enlarged to meet the rapid advances made by the growing science.

Dr. Harris having died in 1860, and the status of the profession demanding that the work be thoroughly revised and enlarged for the ninth edition, Dr. Philip S. Austin, now deceased, was selected to do the work. He was assisted by Prof. F. J. S. Gorgas in the departments of pathology and surgery, and by Dr. Thomas S. Latimer in anatomy and physiology. Each of these men had been associated with Dr. Harris as a teacher in the Baltimore College of Dental Surgery, and under their joint care the tenth edition, of five thousand copies, was issued in 1871. Very much of the work was entirely rewritten, and considerable additions were made to the text.

This large edition sufficed for the wants of the profession for some years, but as the copies upon the shelves of the publishers gradually disappeared, it became evident that yet another edition must be prepared. Since the publication of the tenth edition, wonderful advances had been made in some of the fields of dental science. The histology of 1870 had become a thing of the past. Recent discoveries, by careful observers, had shown that some of the teachings of the tenth edition were erroneous. In pathology the advances had been most marked, and the book had, even in the few years that had elapsed, become almost antiquated. The teachings of 1870 would not answer for 1880, much less for 1885. In mechanics and prosthesis the change in practice had not been so marked, but even here careful revision was necessary. The work of preparing the new edition was entrusted to Prof. F. J. S. Gorgas, one of the editors of the preceding edition, as the man best qualified in all respects for the labor, and the consequence is a book that may almost be called a cyclopedia of dental practice. So great is the contrast with the tenth edition, that it may be considered practically a new book. Much of the matter in the old edition has been struck out as obsolete, or erroneous, yet so large have been the additions that the new work exceeds the size of the old by two hundred pages. Four hundred new illustrations have been added, and many of these are the most valuable that the work contains. To-day, Harris' Principles and Practice is the most complete text-book known to the profession.

In addition to the sale of the former editions in this country, the book has received a large and steady demand from the profession in England, and it has been translated into French. Including the present edition, eighteen thousand five hundred copies have been printed, a most unprecedented sale for a work of this character. The amount paid in royalties to Dr. Harris and his heirs must have amounted to a very large sum, and the copyright money is still paid them.

In writing this sketch of his principal work, it may not be amiss to include a few facts concerning the honored author, for every dentist should know something of the life of one so prominent in his profession.

Chapin A. Harris was born at Pompey, Onondaga Co., N. Y., in 1806. He graduated in medicine in 1830, and for a few years was a general practitioner, when he turned his attention to dentistry. Besides his continual contributions to the *American Journal of Dental Science*, he wrote a "Dictionary of Medicine and Dental Surgery," of which five editions have been published, and he translated from the French a number of works of importance. He died in September, 1860, at the age of fifty-four, from a disease contracted through overwork.

When we set out to write this notice, it was with the intention of presenting a digest of the contents of the eleventh edition of Dr. Harris' great work, and to this end copious notes were made during its examination, and it was carefully compared with the ninth and tenth editions. But limited space forbids the pursuance of this plan, nor is it necessary. It is sufficient to say that the book is brought up fully abreast the advance of thought, and to-day we may take an honest pride in the fact that dentistry possesses such a text-book. Instead of the teachings of Goodsir, and Robertson, and Bell, we have the later discoveries and observations of Kölliker, and Waldeyer, and Miller. In every department it is made complete, and in harmony with the most modern accepted theories. It is an essential in the library of every dental student, and is quite as necessary to the possessors of the earlier editions as to those who have never seen them.

It is needless to say that the publishers have done their full duty, and that the letter press, illustrations and binding, are perfect of their kind.

DENTAL SURGERY for Practitioners and Students. By ASHLY W. BARRETT, M. B., M. R. C. S., L. D. S., Dental Surgeon to the London Hospital. Philadelphia: Presley Blakiston, Son & Co., 1885. Price, $1.00.

The preface of this handbook informs us that it is intended for the use of the busy medical practitioner, who has not the leisure for a more extended study of dental surgery. Medical men have paid but little attention to oral diseases, leaving their consideration and treatment to the dental specialist, but of late American medical colleges have begun the teaching of such general treatment as should fall within the province of every practitioner. Physicians should at least understand enough of dental diseases to prevent the egregious errors into which some of them are betrayed, and this book seems well adapted to the end in view. It does not pretend to teach special dental practice, but the dental student and practitioner will find in it much to interest and instruct them.

ZAHNÄRZTLICHER ALMANACH, 1885. An alphabetical list of all the qualified Dentists practicing within the German Empire, and in Austro-Hungary. Edited by ADOLPH PETERMANN, D. D. S., Frankfort-on-the-Main.

This is an annual which has been issued by Dr. Petermann for the past six years. It contains a great deal of information that is useful to the dental practitioner, and is printed in a very neat form. By it we learn that there are in the German and Austrian Empires but 733 qualified dentists. This does not, however, include the dental artisans, who are allowed to practice mechanical dentistry. There is a list of the principal cities with the names of the qualified practitioners in each, and from it we learn that in the City of Berlin, with its 1,230,000 inhabitants, there are but seventy-five qualified dentists. Dresden, with 240,000 inhabitants, has but sixteen. The book contains excellent portraits of Dr. Suersen, of obturator fame, and Dr. Herbst, the originator of the rotary method of condensing gold foil.

A MOTHER TO MOTHERS. By MRS. M. W. J. Philadelphia: T. B. Welch & Son, 1885.

This is a revised and enlarged edition of the well-known letters published in the *Southern Dental Journal*. We have before this had occasion to commend these excellent essays. The little work should be in the hands of every mother, or expectant mother, and its careful perusal might save an infinite amount of misery, and contribute toward an improved generation of men and women. It may be obtained of the publishers.

THE DIAGNOSIS AND TREATMENT OF NASAL CATARRH. By GEO. M. LEFFERTS, A. M., M. D.

If we have a national disease it is probably the nasal catarrh that is so prevalent and common in many portions of this country. Charles Dickens spoke of himself as having "surprised his friends and delighted his enemies by his truly wonderful performance upon the great American Catarrh," during a visit to this country. There is probably scarcely a reader of this journal who is not interested in the subject, through having suffered, either in person or by a near friend, and who is not anxious to know the best method of treatment. To such we recommend this little work, which may be obtained of Lambert & Co., St. Louis.

PROCEEDINGS OF THE AMERICAN SOCIETY OF MICROSCOPISTS. Seventh Annual Meeting, held in Rochester, August, 1884.

Every one of the annual volumes of proceedings of this society has been of permanent value, and they increase in interest from year to year. The present one contains 300 pages, and presents valuable original papers and much matter of scientific interest. The volume is a handsome one, and reflects credit upon the publication committee. Copies may be obtained of the secretary, Prof. D. S. Kellicott, Buffalo, N. Y.

TRANSACTIONS OF THE AMERICAN DENTAL ASSOCIATION. Twenty-fourth Annual Session. Philadelphia: The S. S. White Dental Mfg. Co., 1885.

This is a handsome volume, and the contents are very well edited, but the matter has become rather stale through the long delay in publishing. Most of the papers and abstracts of the discussions were given to the profession by the journals, months ago. They had their day, but other subjects have superseded them, and the volume now comes upon us like the ghost of things departed. As a permanent record it is of great value, and this, perhaps, should be the principal object sought in its publication. As a contribution to current literature, the journals long ago forestalled it.

We have also received the following books and pamphlets:

*The Present System of Education a Proximate Cause of Dental Decay.* A paper read before the Odontological Society of Pennsylvania. By THEODORE F. CHUPEIN, D. D. S.

A thoughtful and instructive essay that should be read by every practicing dentist.

*Ten Days in the Laboratory of Dr. Robert Koch, of Berlin.* By GEO. W. LEWIS, JR., A. B. Reprinted from the *Buffalo Medical and Surgical Journal.*

*The Treatment of Diseases of the Skin.* A paper read before the section of dermatology and syphilis at the meeting of the International Medical Congress at Copenhagen, Denmark, August, 1884. By JOHN V. SHOEMAKER, A. M., M. D.

*The Oleates.* Further investigation into their Nature and Action. By JOHN V. SHOEMAKER, A. M., M. D.

*Art in Dentistry.* Injurious effects of vulcanized rubber, and other things to be thought of by those compelled to wear artificial teeth. By L. P. HASKELL; Professor of Prosthetic Dentistry in the Chicago College of Dental Surgery.

*Cremation Scientifically and Religiously Considered.* By HENRY HOUSTON BONNELL.

*Outline of Vegetable Histology.* By MRS. WM. STREETER, President of the Section of Botany, R. A. S. Rochester: Davis & Leyden. Price, 50 cents.

*Address in Medicine.* Delivered before the Medical Society of the State of Pennsylvania, at its Annual Meeting held in Philadelphia, May, 1884. By W. H. DALY, M. D.

*Sixteenth Annual Report of the Trustees of the Willard Asylum for the Insane.*

*Annual Report of the Board of Managers of the New York State Reformatory at Elmira.*

*Amended Constitution of the American Dental Association, with the Code of Dental Ethics,* 1885.

*Report of Committee on School Hygiene in Tennessee.* By DANIEL F. WRIGHT, M. D.

*Vital Statistics in Tennessee.* A Report by J. D. PLUNKETT, M. D.

*Extensive Burn, involving the Cavity of the Knee Joint.* By W. H. Daly, M. D.

*Typhoid Fever and Low Water in Wells.* By Henry B. Baker, M. D. Reprinted from the Annual Report of the Michigan State Board of Health.

